# Prevalence of Dizziness and Associated Factors in South Korea: A Cross-Sectional Survey From 2010 to 2012

**DOI:** 10.2188/jea.JE20160113

**Published:** 2018-04-05

**Authors:** Jiwon Chang, Soon Young Hwang, Su Kyoung Park, Jin Hwan Kim, Hyung-Jong Kim, Sung-Won Chae, Jae-Jun Song

**Affiliations:** 1 Department of Otolaryngology Head and Neck Surgery, Hallym University College of Medicine, Seoul, Republic of Korea; 2 Department of Biostatistics, College of Medicine, Korea University, Seoul, Republic of Korea; 3 Department of Otolaryngology Head and Neck Surgery, Korea University College of Medicine, Seoul, Republic of Korea

**Keywords:** dizziness, epidemiology, prevalence, risk factor

## Abstract

**Background:**

Dizziness is one of the most common complaints in medicine and a frequent symptom among older people. Dizziness has a considerable impact on life quality and is associated with high economic costs. The purpose of this study was to evaluate the prevalence of dizziness in the general population and to describe its clinical characteristics and associated factors.

**Methods:**

The Korea National Health and Nutrition Examination Survey (KNHANES) is a cross-sectional survey of the civilian, non-institutionalized population of South Korea. We evaluated data for 12,653 participants (5,450 men and 7,203 women), aged 40 years and above, whom participated in the KNHANES between 2010 and 2012.

**Results:**

In the age group over 40 years old, the 1-year prevalence of dizziness was 20.10%. Dizziness was more prevalent among women (25.18%) than among men (14.57%; *P* < 0.001) and the prevalence rate increased with age (*P* < 0.001). In multivariable analysis, female sex, older age, serum triglyceride level, experience of depression, limited functional status owing to visual acuity impairment, limited physical performance, smoking, alcohol consumption, and perception of stress were independently associated with dizziness.

**Conclusions:**

In our study, the prevalence of dizziness in the general population was 20.10%. There was a stronger relationship between dizziness and physical performance, chronic diseases, and health behaviors compared to that with otologic diseases. Interventions for dizziness should be approached in a multifactorial manner and an understanding of various factors is necessary for the prevention and management of this condition.

## INTRODUCTION

Dizziness is one of the most common complaints in medicine and a frequent symptom in older people^1^; however, it is a vague term describing a wide range of sensations that point in different diagnostic directions. Vertigo is a rotational sense of self or surroundings and is caused by dysfunction of either the peripheral or central vestibular system. A sensation of lightheadedness, unsteadiness, giddiness, or presyncope implies dizziness of non-vestibular origin, such as cardiovascular or neuromuscular dysfunction. The sensation of dizziness itself is an annoying and often intolerable symptom for patients. Moreover, the impact of dizziness appears to be considerable and affects quality of life. Dizziness is related to clinically significant outcomes, such as falls, and is associated with high economic costs.^2^^,^^3^ A study reported that individuals with vestibular vertigo experience interruption in their daily activities, and 80% of participants felt the need for sick leave or medical consultation.^4^ Another study on the social impact of dizziness reported that 27% of participants changed jobs, 21% stopped working, 50% reported reduced efficiency at work, 57% experienced disruption to their social life, 35% experienced family difficulties, and 50% reported difficulties with travel.^5^ Because of the considerable impact of dizziness, it is important to estimate its prevalence in the general population and to identify and address factors related to dizziness.

The prevalence of dizziness ranges from 17% to 30%, depending on the definition used and the population studied.^6^ Various studies concerning the prevalence of dizziness in older populations have been conducted previously.^2^^,^^7^^–^^17^ The lifetime prevalence of dizziness has been estimated as 16.9–23.2%.^7^^,^^13^ Studies investigating dizziness in the past month have reported a prevalence of 15.8–23.0%^2^^,^^18^ and those investigating dizziness in the past 1 year have reported a prevalence of 10.9–59.2%.^12^^,^^19^

The present study was conducted to determine the national prevalence of dizziness in South Korea based on survey data obtained from the 2010 to 2012 Korea National Health and Nutrition Examination Survey (KNHANES) and to investigate associations between demographic, otologic, medical, functional, behavioral, and psychosocial factors related to dizziness. Identification and modification of these factors may help to reduce the incidence of dizziness.

## METHODS

### Study population and data collection

Nationwide epidemiologic studies conducted by government organizations can provide robust data for investigating the national prevalence of disease conditions. The KNHANES was begun in 1998 to examine the general health and nutrition status of the population of South Korea. Every year, 10,000 to 12,000 individuals in about 4,600 households are selected from a panel to represent the national population using a multistage clustered and stratified random sampling method that is based on the National Census Data. The participation rate among the selected households in the past several cycles of KNHANES has been high, ranging from 79% to 84%.^20^ In KNHANES V (from 2010 to 2012), 576 communities (11,520 households) were selected, and people residing in these communities were randomly selected based on the National Census Data. The response rate (the number of respondents divided by the number of people resided in the selected community) for KNHANES V was 80.8%.

Among 25,534 individuals who participated in the KNHANES from 2010 to 2012 (representing 49,093,627 individuals in South Korea), a total of 12,653 individuals aged 40 years and older (representing 22,696,166 individuals in that age group in South Korea) were included in our study. Of these, 5,450 were men and 7,203 were women. Written informed consent was obtained from all participants prior to the survey, and approval for this research was obtained from the Institutional Review Board of Korea University Guro Hospital (KUGH14304-001).

### Definition of dizziness

Participants aged 40 years or older were asked about their experiences of dizziness. If the participant had experienced dizziness or imbalance during the past 12 months, they were considered to have dizziness. That is, participants with positional vertigo at the time of the survey and who had experienced such movement-induced vertigo within the past 1 year were all included in the dizziness group. Participants who had an imbalance or tendency to fall without an external stimulus were also included in the dizziness group.

### Evaluation of associated factors

Participants were interviewed regarding their health and nutrition. The questionnaire addressed demographic and socioeconomic characteristics, physical health, chronic diseases, lifestyle behaviors, health-related quality of life, and health care utilization. Participants were also asked to undergo a basic health assessment that included blood and urine sample testing; blood pressure (BP) measurement; pulmonary function testing; and ophthalmologic, dental, and otolaryngologic examinations. To determine the incidence of otologic diseases, such as tympanic membrane (TM) perforation, cholesteatoma, and otitis media, all participants underwent an ear examination using a 4-mm 0°-angled rigid endoscope attached to a charge-coupled device (CCD) camera.

Demographic data included age, sex, and household income (grouped as lower, lower-middle, upper-middle, and upper income levels). Physical examination included waist circumference (cutoff values were 90 cm for men and 80 cm for women), serum triglyceride (TG) level (increased serum TG level was defined as ≥150 mg/dL), serum high-density lipoprotein (HDL) cholesterol level (low serum HDL level was defined as ≤40 mg/dL for men and ≤50 mg/dL for women), and serum vitamin D level. Presence of the following chronic diseases or disorders was ascertained through the interviews and physical examination: hypertension (defined as systolic/diastolic BP >120/80 mm Hg), diabetes (defined as fasting blood glucose level >126 mg/dL), history of cerebral stroke, myocardial infarction, chronic obstructive pulmonary disease (COPD), arthritis, and depression. We evaluated data from the survey and otolaryngologic examination for the presence of TM perforation, cholesteatoma, otitis media with effusion, or chronic otitis media. In addition, smoking status (current, former, or never smoker), alcohol consumption (consumption of alcohol more than once per month), sleep duration (≤5, 6, 7, 8, or ≥9 hours per day), and perceived stress (very much, much, little, or none) were estimated. In addition, functional status or activities of daily living (ADL) was evaluated and categorized as follows: limited functional status owing to visual acuity impairment (yes/no), limited physical performance (no difficulty walking, partial difficulty walking, or bedridden), and limited activities of daily living (no difficulty with ADL, partial difficulty with ADL, or cannot perform ADL).

### Statistical analysis

We calculated the prevalence and 95% confidence intervals (CIs) for dizziness, and potentially associated factors were evaluated by univariable analysis. Only variables with *P*-value ≤0.05 were selected for multivariable analysis in the logistic regression model. In univariable analysis, the Rao-Scott chi-square test using PROC SURVEYFREQ in SAS version 9.3 (SAS Institute Inc., Cary, NC, USA), and logistic regression analysis using PROC SURVEYLOGISTIC in SAS, were used to test the association between dizziness and risk factors in a complex sampling design. In multivariable analysis, adjusted odds ratios (ORs) with 95% CIs were calculated using logistic regression analysis (PROC SURVEYLOGISTIC in SAS). To reflect national population estimates, sample weights were applied in all analyses. All *P*-values were two-sided, and *P* < 0.05 was considered to be statistically significant.

## RESULTS

### Prevalence of dizziness

Among the 12,653 participants, 2,743 had experienced episodes of dizziness during the past 1 year, and the prevalence was 20.10% (95% CI, 18.93–21.27%). The prevalence of dizziness was higher among women (25.18%; 95% CI, 23.65–26.71%) than among men (14.57%; 95% CI, 13.19–15.96%) (*P* < 0.001). Dizziness increased with age (*P* < 0.001), starting with 14.05% (95% CI, 12.50–15.59%) in the age group of 40 years and older and reaching 37.57% (95% CI, 32.78–42.36%) in the group aged over 80 years (Table 1).

**Table 1.** Prevalence of dizziness by sex and age group in participants over 40 years old (*n* = 12,653)

Age, years	Male		Female		Total	
		
	*N*^a^	Prevalence (95% CI)^b^	*N*	Prevalence (95% CI)	*N*	Prevalence (95% CI)
40–49	1,388	10.17 (8.18–12.16)	1,784	18.05 (15.90–20.20)	3,172	14.05 (12.50–15.59)
50–59	1,427	11.95 (9.89–14.00)	2,000	21.50 (19.13–23.87)	3,427	16.76 (15.09–18.43)
60–69	1,387	18.96 (16.11–21.81)	1,698	31.30 (28.22–34.38)	3,085	25.42 (23.12–27.71)
70–79	1,056	25.50 (22.11–28.89)	1,365	37.76 (34.16–41.37)	2,421	32.79 (30.03–35.55)
>80	192	38.13 (29.59–46.78)	356	37.32 (31.21–43.42)	548	37.57 (32.78–42.36)

Total	5,450	14.57 (13.19–15.96)	7,203	25.18 (23.65–26.71)	12,653	20.10 (18.93–21.27)

CI, confidence interval.

^a^Number of participants over 40 years old.

^b^Prevalence and 95% CI of dizziness in same sex, age group.

The prevalence of dizziness in men increased continuously with age, from 10.17% (95% CI, 8.18–12.16) for men in their 40s, 11.95% (95% CI, 9.89–14.00%) in their 50s, 18.96% (95% CI, 16.11–21.81%) in their 60s, 25.45% (95% CI, 22.11–28.89%) in their 70s, and 38.13% (95% CI, 29.59–46.68%) in the group aged 80 years and older. Prevalence among women also increased with age: 18.05% (95% CI, 15.90–20.20%) for women in their 40s, 21.50% (95% CI, 19.13–23.87%) in their 50s, 31.30% (95% CI, 28.22–34.38%) in their 60s, 37.76% (95% CI, 34.16–41.37%) in their 70s, and 37.32% (95% CI, 31.21–43.42%) in their 80s or older (Table 1).

### Analysis of associated factors

Experiences of dizziness and its associated factors were investigated using univariable and multivariable analyses. When we analyzed various factors without sex stratification, dizziness was more prevalent among women than among men (OR 1.97; 95% CI, 1.75–2.22 in univariable analysis; OR 1.69; 95% CI, 1.43–1.98 in multivariable analysis), so we investigated separately for men and women. Univariable analysis demonstrated that, in men, dizziness was associated with age, household income, serum vitamin D, history of cerebral stroke, arthritis, experience of depression, limited functional status owing to visual acuity impairment, limited physical performance, limited ADL, smoking, and perception of stress. No otolaryngologic problems, such as TM perforation, cholesteatoma, otitis media with effusion, or chronic otitis media, were associated with dizziness (Table 2). In women, dizziness was associated with age, household income, serum TG level, history of cerebral stroke, myocardial infarction, arthritis, experience of depression, limited functional status owing to visual acuity impairment, limited physical performance, limited ADL, smoking, alcohol consumption, sleep duration, and perception of stress (Table 2).

**Table 2.** Analysis of associated factors in men over 40 years old (*n* = 5,450)

Variables	N (%)^a^ (95% CI)	Dizziness^b^ (95% CI)	Univariable analysis		Multivariable analysis^d^	
	
			*P* value	OR (95% CI)	*P* value	OR (95% CI)
**Demographic data**						
Age, years (5,450)^c^			0.00		0.00	
40–49	37.99 (36.02–39.97)	10.17 (8.18–12.16)		1		1
50–59	31.19 (29.56–32.83)	11.95 (9.89–14.00)		1.19 (0.91–1.57)		1.22 (0.90–1.64)
60–69	17.73 (16.58–18.87)	18.96 (16.11–21.81)		2.07 (1.57–2.73)		2.11 (1.54–2.89)
70–79	10.81 (9.94–11.69)	25.45 (22.11–28.89)		3.02 (2.31–3.96)		2.55 (1.82–3.57)
>80	2.27 (1.87–2.67)	38.13 (29.59–46.68)		5.45 (3.57–8.31)		5.26 (2.90–9.55)
House income (5,374)			0.04		0.93	
Lower (%)	18.14 (16.80–19.49)	23.28 (20.10–26.47)		1.44 (1.07–1.94)		0.99 (0.73–1.36)
Lower-middle (%)	26.39 (24.73–28.06)	13.85 (11.511–16.19)		1.03 (0.79–1.35)		0.92 (0.68–1.23)
Upper-middle (%)	26.98 (25.42–28.54)	12.19 (9.89–14.49)		1 (0.75–1.33)		0.94 (0.70–1.27)
Upper (%)	28.48 (26.64–30.33)	11.87 (9.78–13.95)		1		1

**Physical examination**						
Waist circumference (5,430)			0.71			
<90 cm	72.21 (70.63–73.78)	14.34 (12.75–15.93)		1		
≥90 cm	27.79 (26.22–29.37)	15.11 (12.72–17.51)		1.04 (0.83–1.31)		
Serum TG level (5.035)			0.13			
<150 mg/dL	54.30 (52.55–56.04)	13.73 (12.06–15.40)		1		
≥150 mg/dL	45.70 (43.96–47.45)	14.48 (12.43–16.52)		1.17 (0.96–1.44)		
Serum HDL level (5,066)			0.12			
>40 mg/dL	67.33 (65.70–68.95)	13.92 (12.30–15.53)		1		
≤40 mg/dL	32.67 (31.05–34.30)	14.72 (12.59–16.84)		1.04 (0.84–1.23)		
Vitamin D (5,450)			0.02	0.98 (0.96–0.99)	0.06	0.98 (0.97–1.00)

**Otologic examination**						
TM perforation (No)	96.86 (96.26–97.46)	13.99 (12.61–15.37)	0.41	1		
(5,009) (Yes)	3.14 (2.54–3.74)	19.42 (11.98–26.86)		1.22 (0.76–1.99)		
Cholesteatoma (No)	97.85 (97.33–98.36)	14.13 (12.74–15.53)	0.99	1		
(5,009) (Yes)	2.15 (1.64–2.67)	15.36 (7.69–23.02)		1.00 (0.55–1.81)		
Otitis media with effusion (No)	99.30 (99.00–99.59)	14.10 (12.07–15.50)	0.55	1		
(5,009) (Yes)	0.70 (0.41–1.00)	22.66 (6.41–38.91)		1.36 (0.50–3.72)		
Chronic otitis media (No)	95.13 (94.35–95.90)	14.00 (12.62–15.39)	0.70	1		
(5,009) (Yes)	4.87 (4.10–5.64)	17.19 (11.53–22.85)		1.08 (0.73–1.61)		

**Comorbidities**						
Hypertension (5,253)^c^			0.88			
No	45.13 (43.38–46.88)	13.47 (11.60–15.34)		1		
Yes	54.87 (53.12–56.62)	15.07 (13.26–16.88)		0.98 (0.80–1.21)		
DM (5,035)			0.46			
No	76.13 (74.59–77.67)	13.43 (11.90–14.96)		1		
Yes	23.87 (22.33–25.41)	16.12 (13.46–18.78)		1.11 (0.87–1.41)		
Cerebral stroke (5,268)			0.00		0.09	
No	97.55 (97.11–97.99)	13.90 (12.52–15.28)		1		1
Yes	2.45 (2.01–2.89)	31.51 (24.10–38.92)		1.85 (1.29–2.66)		1.41 (0.95–2.11)
Myocardial infarction (5,268)			0.43			
No	96.33 (95.74–96.92)	14.07 (12.67–15.46)		1		
Yes	3.67 (3.08–4.26)	21.31 (14.61–28.01)		1.20 (0.77–1.86)		
Chronic obstructive pulmonary disease (5,106)			0.75			
No	99.07 (98.77–99.36)	14.33 (12.93–15.73)		1		
Yes	0.93 (0.64–1.23)	20.65 (6.66–34.65)		1.15 (0.49–2.67)		
Arthritis (5,268)			0.00		0.05	
No	91.12 (90.24–92.01)	13.28 (11.89–14.66)		1		1
Yes	8.88 (7.99–9.76)	25.16 (20.63–29.69)		1.73 (1.32–2.26)		1.38 (1.00–1.92)
Depression (5,268)			0.00		0.00	
No	91.38 (90.46–92.30)	13.16 (11.78–14.54)		1		1
Yes	8.62 (7.70–9.54)	26.74 (21.42–32.06)		2.32 (1.73–3.10)		1.71 (1.24–2.36)

**Functional status**						
Limited functional status due to visual acuity (5,265)			0.00		0.03	
No limitation	89.35 (88.28–90.43)	12.82 (11.43–14.21)		1		1
Limitation due to visual acuity	0.83 (0.52–1.13)	22.99 (10.63–35.34)		1.70 (0.88–3.26)		1.39 (0.69–2.80)
Limitation but no due to visual acuity	9.82 (8.79–10.86)	27.24 (22.40–32.07)		2.09 (1.58–2.77)		1.56 (1.11–2.19)
Physical performance (5,265)			0.00		0.04	
No difficulties in walking	86.87 (85.75–87.98)	12.08 (10.72–13.44)		1		1
Partial difficulties in walking	12.61 (11.53–13.70)	28.08 (24.05–32.11)		2.12 (1.68–2.68)		1.45 (1.05–2.01)
Bedridden	0.52 (0.30–0.74)	54.01 (32.80–75.22)		5.17 (1.91–14.00)		2.71 (0.81–9.11)
Activity of daily living (5,265)			0.00		0.96	
No difficulties in ADL	91.53 (90.57–92.49)	12.79 (11.41–14.17)		1		1
Partial difficulties in ADL	7.44 (6.57–8.30)	29.71 (24.31–35.12)		2.20 (1.64–2.97)		1.01 (0.69–1.49)
Cannot do ADL	1.04 (0.71–1.36)	38.59 (23.63–53.56)		3.05 (1.68–5.55)		1.13 (0.48–2.66)

**Health behavior**						
Smoking (5,266)			0.02		0.07	
No	58.82 (57.14–60.50)	14.12 (12.54–15.69)		1		1
Yes	41.18 (39.50–42.86)	14.49 (12.48–16.49)		1.25 (1.03–1.50)		1.2 (0.98–1.46)
Alcohol consumption (once a month) (5,248)			0.12			
No	27.38 (25.85–28.91)	17.49 (15.01–19.97)		1		
Yes	72.62 (71.09–74.15)	13.10 (11.59–14.61)		0.85 (0.69–1.05)		
**Sleep hours** (5,256)			0.44			
≤5 hr	13.30 (12.17–14.43)	18.20 (14.86–21.53)		1.20 (0.97–1.59)		
≤6 hr	27.76 (26.18–29.34)	13.46 (11.29–15.62)		1.03 (0.79–1.35)		
≤7 hr	29.43 (27.81–31.04)	13.02 (10.84–15.20)		1		
≤8 hr	22.22 (20.83–23.61)	13.44 (10.78–16.10)		0.93 (0.69–1.25)		
≥9 hr	7.30 (6.37–8.22)	17.89 (13.03–22.75)		1.19 (0.82–1.74)		

**Perception of stress** (5,266)			0.00		0.00	
Very much	3.39 (2.79–3.98)	18.85 (12.16–25.54)		2.05 (1.25–3.35)		1.44 (0.85–2.43)
Much	18.00 (16.69–19.30)	18.67 (15.63–21.70)		2.30 (1.65–3.21)		1.72 (1.21–2.44)
Little	60.95 (59.40–62.50)	12.74 (11.04–14.44)		1.26 (0.96–1.65)		1.13 (0.85–1.49)
None	17.67 (16.42–18.92)	14.18 (11.55–16.81)		1		1

ADL, activities of daily living; CI, confidence interval; DM, diabetes mellitus; HDL, high-density lipoprotein; OR, odds ratio; TG, triglycerides; TM, tympanic membrane.

^a^Proportion of participants in the same group.

^b^Prevalence of dizziness in the subgroup.

^c^Numbers in parentheses: number of participants included in that particular factor.

^d^Variables with *P*-value ≤0.05 in univariable analysis were selected.

Adjusted for age.

After multivariable-adjusted analysis in men, older age (OR 5.26; 95% CI, 2.90–9.55 for those in their 80s and older; OR 2.55; 95% CI, 1.82–3.57 in their 70s; and OR 2.11; 95% CI, 1.54–2.89 in their 60s), experience of depression (OR 1.71; 95% CI, 1.24–2.36), limited functional status owing to impairment of visual acuity (OR 1.39; 95% CI, 0.69–2.80), limited physical performance (OR 1.45; 95% CI 1.05–2.01 in participants with partial difficulties in walking; OR 2.71; 95% CI, 0.81–9.11 in bedridden participants), and perception of stress (OR 1.72; 95% CI, 1.21–2.44 in participant with ‘much’ perception of stress) were associated with dizziness (Table 2). In women, multivariable analysis demonstrated that older age (OR 1.36; 95% CI, 0.86–2.14 for those in their 80s and older; OR 1.54; 95% CI, 1.16–2.04 in their 70s; and OR 1.40; 95% CI, 1.09–1.79 in their 60s), higher serum TG level (OR 1.28; 95% CI, 1.09–1.50), experience of depression (OR 1.23; 95% CI, 1.03–1.47), limited functional status owing to impairment of visual acuity (OR 1.32; 95% CI, 1.04–1.69), limited physical performance (OR 1.35; 95% CI 1.09–1.67 in participants with partial difficulties in walking; OR 2.12; 95% CI, 1.09–4.12 in bedridden participants), smoking (OR 1.61; 95% CI, 1.15–2.27), alcohol consumption (OR 0.85; 95% CI, 0.73–0.99), and perception of stress (OR 1.88; 95% CI, 1.31–2.69 in participant with ‘very much’ perception of stress; OR 1.48; 95% CI, 1.17–1.86 in participant with ‘much’ perception of stress) were associated with dizziness (Table 3).

**Table 3.** Analysis of associated factors in women over 40 years old (*n* = 7,203)

Variables	*N* (%)^a^ (95% CI)	Dizziness^b^ (95% CI)	Univariable analysis		Multivariable analysis^d^	
	
			*P* value	OR (95% CI)	*P* value	OR (95% CI)
**Demographic data**						
Age, years (7,203)^c^			0.00		0.02	
40–49	33.85 (32.18–35.51)	18.05 (15.90–20.20)		1		1
50–59	29.11 (27.71–30.51)	21.50 (19.13–23.87)		1.24 (1.03–1.51)		1.10 (0.88–1.36)
60–69	17.86 (16.83–18.87)	31.30 (28.22–34.38)		2.07 (1.70–2.52)		1.40 (1.09–1.79)
70–79	14.55 (13.55–15.56)	37.76 (34.16–41.37)		2.76 (2.24–3.39)		1.54 (1.16–2.04)
>80	4.63 (4.00–5.26)	37.32 (31.21–43.42)		2.70 (2.00–3.66)		1.36 (0.86–2.14)
House income (7,099)			0.00		0.26	
Lower (%)	24.86 (23.33–26.39)	36.35 (33.40–39.30)		1.56 (1.27–1.91)		1.15 (0.92–1.45)
Lower-middle (%)	27.27 (25.88–28.65)	23.86 (21.21–26.50)		1.11 (0.90–1.37)		1.00 (0.79–1.25)
Upper-middle (%)	23.27 (21.96–24.58)	20.16 (17.54–22.78)		0.96 (0.77–1.20)		0.9 (0.71–1.14)
Upper (%)	24.60 (23.07–26.13)	20.27 (17.81–22.74)		1		1

**Physical examination**						
Waist circumference (7,178)			0.72			
<80 cm	48.59 (46.94–50.24)	23.87 (21.91–25.83)		1		
≥80 cm	51.41 (49.76–53.06)	26.47 (24.45–28.49)		0.97 (0.85–1.12)		
Serum TG level (6,538)			0.00		0.00	
<150 mg/dL	66.25 (64.77–67.72)	21.36 (19.66–23.06)		1		1
≥150 mg/dL	33.75 (32.27–35.23)	29.70 (26.96–32.44)		1.34 (1.14–1.58)		1.28 (1.09–1.50)
Serum HDL level (6,571)			0.98			
>50 mg/dL	46.52 (44.96–48.08)	23.22 (21.17–25.28)		1		
≤50 mg/dL	53.48 (51.92–55.04)	25.23 (23.23–27.23)		1.00 (0.87–1.15)		
Vitamin D (7,203)			0.06	0.99 (0.98–1.00)		

**Otologic examination**						
TM perforation (No)	96.61 (96.04–97.18)	24.56 (22.99–26.14)	0.78	1		
(6,659) (Yes)	3.39 (2.82–3.96)	26.47 (19.27–33.66)		0.95 (0.65–1.39)		
Cholesteatoma (No)	97.25 (96.65–97.85)	24.38 (22.82–25.94)	0.054	1		
(6,659) (Yes)	2.75 (2.15–3.34)	33.32 (25.27–41.37)		1.43 (0.99–2.06)		
Otitis media with effusion (No)	99.23 (98.91–99.55)	24.53 (22.97–26.09)	0.22	1		
(6,659) (Yes)	0.77 (0.45–1.09)	37.10 (20.60–53.59)		1.59 (0.75–3.38)		
Chronic otitis media (No)	94.20 (93.41–94.99)	24.29 (22.72–25.87)	0.22	1		
(6,659) (Yes)	5.80 (5.01–6.59)	30.04 (24.29–35.80)		1.19 (0.9–1.58)		

**Comorbidities**						
Hypertension (6.981)^c^			0.24			
No	52.79 (51.01–54.56)	21.27 (19.47–23.07)		1		
Yes	47.21 (45.44–48.99)	29.11 (26.78–31.45)		1.10 (0.94–1.26)		
DM (6,539)			0.72			
No	83.13 (81.95–84.32)	23.40 (21.77–25.04)		1		
Yes	16.87 (15.68–18.05)	28.05 (24.58–31.53)		1.03 (0.86–1.25)		
Cerebral stroke (7,003)			0.04		0.25	
No	97.94 (97.54–98.34)	24.66 (23.11–26.20)		1		1
Yes	2.06 (1.66–2.46)	39.15 (30.27–48.03)		1.49 (1.02–2.17)		1.28 (0.84–1.97)
Myocardial infarction (7,003)			0.01		0.07	
No	96.61 (96.10–97.13)	24.42 (22.87–25.97)		1		1
Yes	3.39 (2.87–3.90)	40.26 (32.58–47.94)		1.6 (1.15–2.24)		1.39 (0.97–1.97)
Chronic obstructive pulmonary disease (6,596)			0.23			
No	99.78 (99.64–99.91)	24.49 (22.94–26.04)		1		
Yes	0.22 (0.09–0.36)	13.10 (0.00–28.73)		0.42 (0.10–1.73)		
Arthritis (7,001)			0.01		0.53	
No	71.98 (70.66–73.31)	22.36 (20.71–24.00)		1		1
Yes	28.02 (26.69–29.34)	31.64 (29.09–34.18)		1.21 (1.05–1.41)		0.95 (0.80–1.13)
Depression (7,001)			0.00		0.02	
No	78.14 (76.94–79.35)	22.77 (21.19–24.35)		1		1
Yes	21.86 (20.65–23.06)	32.74 (29.63–35.85)		1.66 (1.42–1.92)		1.23 (1.03–1.47)

**Functional status**						
Limited functional status due to visual acuity (6,995)			0.00		0.04	
No limitation	85.40 (84.33–86.47)	22.13 (20.60–23.66)		1		1
Limitation due to visual acuity	0.66 (0.437–0.89)	40.88 (24.39–57.36)		2.06 (0.97–4.37)		1.72 (0.80–3.68)
Limitation but no due to visual acuity	13.93 (12.87–15.00)	41.30 (37.04–45.56)		2.03 (1.67–2.47)		1.32 (1.04–1.69)
Physical performance (6,995)			0.00		0.01	
No difficulties in walking	75.40 (74.11–76.69)	20.46 (18.85–22.06)		1		1
Partial difficulties in walking	23.44 (22.14–24.74)	37.38 (34.26–40.49)		1.82 (1.54–2.15)		1.35 (1.09–1.67)
Bedridden	1.16 (0.85–1.47)	63.95 (52.26–75.63)		4.86 (2.91–8.13)		2.12 (1.09–4.12)
Activity of daily living (6,990)			0.00		0.18	
No difficulties in ADL	85.15 (84.13–86.16)	21.85 (20.33–23.37)		1		1
Partial difficulties in ADL	12.94 (11.97–13.91)	40.69 (36.40–44.99)		1.91 (1.59–2.30)		1.18 (0.92–1.52)
Cannot do ADL	1.91 (1.55–2.27)	54.68 (44.44–64.91)		3.00 (1.96–4.58)		1.55 (0.88–2.71)

**Health behavior**						
Smoking (6,981)			0.00		0.00	
No	95.26 (94.63–95.89)	24.33 (22.76–25.91)		1		1
Yes	4.74 (4.11–5.37)	36.16 (29.75–42.58)		1.90 (1.42–2.55)		1.61 (1.15–2.27)
Alcohol consumption (once a month) (6,958)			0.00		0.04	
No	66.71 (65.33–68.09)	27.37 (25.54–29.19)		1		1
Yes	33.29 (31.91–34.67)	20.09 (17.96–22.23)		0.80 (0.70–0.93)		0.85 (0.73–0.99)
**Sleep hours** (6,961)			0.00		0.05	
≤5 hr	21.20 (20.05–22.34)	32.99 (29.71–36.27)		1.58 (1.29–1.93)		1.36 (1.09–1.68)
≤6 hr	26.57 (25.32–27.82)	22.76 (20.29–25.22)		1.12 (0.92–1.36)		1.10 (0.89–1.36)
≤7 hr	25.94 (24.65–27.22)	20.55 (18.21–22.88)		1		1
≤8 hr	20.12 (18.99–21.24)	23.38 (20.37–26.38)		1.18 (0.96–1.45)		1.16 (0.93–1.45)
≥9 hr	6.18 (5.44–6.92)	29.75 (24.22–35.28)		1.43 (1.05–1.94)		1.36 (0.99–1.86)

**Perception of stress** (6,976)			0.00		0.00	
Very much	4.97 (4.35–5.60)	39.54 (33.11–45.96)		2.63 (1.89–3.66)		1.88 (1.31–2.69)
Much	21.82 (20.62–23.02)	31.10 (28.07–34.12)		1.86 (1.51–2.28)		1.48 (1.17–1.86)
Little	56.79 (55.39–58.19)	21.82 (20.05–23.59)		1.21 (1.99–1.47)		1.13 (0.92–1.39)
None	16.41 (15.36–17.47)	22.88 (19.89–25.86)		1		1

ADL, activities of daily living; CI, confidence interval; DM, diabetes mellitus; HDL, high-density lipoprotein; OR, odds ratio; TG, triglycerides; TM, tympanic membrane.

^a^Proportion of participants in the same group.

^b^Prevalence of dizziness in the subgroup.

^c^Numbers in parentheses: number of participants included in that particular factor.

^d^Variables with *P*-value ≤0.05 in univariable analysis were selected.

Adjusted for age.

## DISCUSSION

Dizziness is a common symptom among the general population and can present as sensations of vertigo, lightheadedness, unsteadiness, disequilibrium, or fainting. These symptoms arise owing to abnormal signals from sensory systems, such as the visual, proprioceptive, and vestibular systems. Since the impact of dizziness appears to be considerable, it is important to identify its prevalence and related factors in the general population.

The prevalence of dizziness in the general population aged 40 years or older during the past 1 year was 20.10% in our study, which is a figure similar to others found in the published literature. Agrawal and colleagues reported that, among 6,785 American participants aged 40 years and above, 27.0% self-reported experiencing dizziness in the prior 12 months.^21^ Various cross-sectional survey studies from Europe have reported the prevalence of self-reported dizziness in the adult population to be from 13% to 28.7%.^18^^,^^22^^,^^23^ These differences in reported rates may be explained by variations in the age groups studied or number of participants enrolled in the surveys. In the present study, the dizziness rate increased substantially with age: participants in their 80s and older (OR 2.28; 95% CI, 1.57–3.32) were more susceptible to dizziness than those in their 70s (OR 1.91; 95% CI, 1.53–2.28); participants in their 70s were more affected than those in their 60s (OR 1.67; 95% CI, 1.38–2.04); and participants in their 60s were more influenced than those in their 50s (OR 1.16; 95% CI, 0.96–1.38) (data not shown). This finding demonstrates that the prevalence of dizziness increases with age and also highlights that variations in age groups would affect prevalence in the general population. The association of dizziness with sex has been observed in some studies^6^^,^^8^ but not in others.^10^^,^^24^ However, in the present study, the prevalence of dizziness during the past year was higher in women (25.18%) than in men (14.57%) (OR 1.68; 95% CI, 1.44–1.98). The differences across studies may be explained by differences in the duration of dizziness or population characteristics.

There were strong associations between dizziness and self-reported functional status variables, as reflected in physical performance, limited functional status owing to impairment of visual acuity, and restrictions in ADL (*P* < 0.001). Self-reported poor health status and mobility have been considered strong medical indicators of health problems and often play an important role in dizziness among older people.^15^^,^^24^ This leads to the assumption that dizziness is often caused by multifactorial medical and functional conditions. Interventions for dizziness should therefore be approached in a multifactorial manner.

Perception of stress was strongly associated with dizziness in the present study. Stress is mental, emotional, or physical strain and tension. Perceived stress was rated as very much, much, little, and none in the survey. Women participants who complained of “very much” stress had an OR for dizziness of 1.88 (95% CI, 1.31–2.69) compared with those who reported no stress. Male participants had similar results, with participants who complained of “much” stress having an OR for dizziness of 1.72 (95% CI, 1.21–2.44). Depression was also related to dizziness, with an OR 1.71 (95% CI, 1.24–2.36) in men and OR 1.23 (95% CI, 1.03–1.47) in women, compared with no history of depression. The association of dizziness with psychological distress has been reported in other studies; however, the question remains unresolved whether dizziness is caused by psychological factors or whether the psychological manifestation is as a result of dizziness. Nevertheless, a vicious circle of interaction seems to exist between dizziness and psychological distress. Orji reported that vertigo attacks in Ménière’s disease produce and increase the level of anxiety and aggravate emotional states via the autonomic nervous system and elevated levels of stress-related hormone.^25^ Other authors have reported that brief dizziness in benign paroxysmal positional vertigo is caused by otolithic dysfunction, but the persistence of dizziness in the disease is correlated with mental stress.^26^ Some studies have suggested that the association of dizziness with psychological distress can be explained via socioeconomic pathways because psychological symptoms are related to disadvantaged social environments, such as poor education, insufficient income, a high rate of unemployment, lower social status, and social insecurity.^24^ In a longitudinal study on the relationship between stress and dizziness, Andersson and Yardley found that mental and emotional stress showed associations with dizziness, whereas physical stress did not.^27^ Also, in our study, short sleep hours (less than 5 hours) were related to dizziness in women (OR 1.36; 95% CI, 1.09–1.68). Sugaya et al reported that the prevalence of sleep disturbance was higher in women dizziness patients, and that the presence of sleep disturbance was associated with severe anxiety and depression and low health-related quality of life.^28^

Lifestyle-related diseases, such high blood pressure, diabetes mellitus, visceral obesity, and dyslipidemia, are considered to be independent risk factors for arteriosclerotic disease. It has recently been suggested that the accumulation of these risk factors increases the risk of cardiovascular disease or cerebral infarction, a theory that is referred to as metabolic syndrome.^29^^,^^30^ In the present study, those with elevated serum TG level (>150 mg/dL) had an OR of 1.28 (95% CI, 1.09–1.50), but both waist circumference and serum HDL level were not related to dizziness.

The associations between chronic conditions and dizziness have been reported previously^31^; in the present study, the bivariate correlation between cerebral infarction and dizziness or-myocardial infarction and dizziness or arthritis and dizziness was significant and strong, but these were not independently associated in multivariate analysis. However, hypertension, diabetes, and COPD were not statistically associated with dizziness in the current study. The relationship between hypertension and dizziness is controversial because this symptom could be related to antihypertensive medication rather than to hypertension itself.^1^^,^^15^ In our analysis, participants with an average of three systolic/diastolic BP measurements higher than 120/80 mm Hg were considered to have hypertension. Because only the BP at the time of measurement was adopted for the analysis, the past diagnosis and duration of hypertension and the effect of antihypertensive medication were not considered. In addition, participants with a fasting blood glucose level higher than 126 mg/dL were considered to have diabetes in our study; the diagnostic status, severity, and duration of disease, and the effect of medication were not included in the analysis.

Health behavior variables (smoking and alcohol consumption) showed interesting results. According to the present study, current smokers reported 1.20 times more dizziness than past-smokers or never smokers (OR 1.20; 95% CI, 0.98–1.46 in men; OR 1.61; 95% CI, 1.15–2.27 in women). Smoking was associated with dizziness in some previous studies^32^^,^^33^ but not in others.^1^^,^^34^ The present results should be interpreted carefully because the smoking duration and amounts of past smokers were not considered and only current smoking status was considered in the analysis. Alcohol consumption in women had an association with dizziness in our analysis. Female participants who consumed more than one alcoholic beverage in a month were less affected by dizziness (OR 0.88; 95% CI, 0.77–1.00) than those who consumed less than one or no alcoholic beverages in a month. This result should be interpreted with caution because in the current analysis, we did not subdivide the amount of alcohol ingested each month.

Eustachian tube dysfunction, middle ear effusion, and otitis media have been considered common causes of balance disturbance in children.^35^^,^^36^ Moreover, the vestibular system is known to be significantly affected in cases of chronic otitis media^37^ and both semicircular canals and the saccule are reported to be influenced in longstanding otitis media. In our study, the OR of otitis media with effusion was 1.50 (1.36 in men and 1.59 in women) in univariable analysis, implying a possible association with dizziness; however, this was not statistically significant. The reason for this might be that the sample size was not sufficiently large to statistically detect the difference. Moreover, in this study, other otologic diseases, such TM perforation, cholesteatoma, and chronic otitis media, were not significantly associated with dizziness.

The strength of our study is that it is an analysis of a large, population-based, nationally representative sample of adults investigating the prevalence and associated factors of dizziness. However, there are some limitations. First, because it is a cross-sectional study, we cannot identify a causal relationship between dizziness and other factors. Second, because our study used certain data from self-reported questionnaires to examine participant lifestyle, recall bias may exist. Third, we could not distinguish between dizziness and vertigo in the study.

### Conclusion

In this study, the 1-year prevalence of dizziness was 20.10% in the general population. Dizziness was more frequent among women, and the prevalence increased with age. Multivariable analysis demonstrated that dizziness is associated with female sex, older age, serum TG level, experience of depression, limited functional status owing to visual acuity impairment, limited physical performance, smoking, alcohol consumption, and perception of stress. This study demonstrates that dizziness is often caused by multifactorial medical and functional conditions. Therefore, interventions for dizziness should be approached in a multifactorial manner. Better understanding and modification of the factors associated with dizziness are required for prevention and management of this condition.
